# Inactivation of Target RNA Cleavage of a III-B CRISPR-Cas System Induces Robust Autoimmunity in *Saccharolobus islandicus*

**DOI:** 10.3390/ijms23158515

**Published:** 2022-07-31

**Authors:** Yan Zhang, Jinzhong Lin, Xuhui Tian, Yuan Wang, Ruiliang Zhao, Chenwei Wu, Xiaoning Wang, Pengpeng Zhao, Xiaonan Bi, Zhenxiao Yu, Wenyuan Han, Nan Peng, Yun Xiang Liang, Qunxin She

**Affiliations:** 1 Henan Engineering Laboratory for Bioconversion Technology of Functional Microbes, College of Life Sciences, Henan Normal University, Xinxiang 453007, China; zhang0516yan@163.com; 2 State Key Laboratory of Agricultural Microbiology, College of Life Science and Technology, Huazhong Agricultural University, Wuhan 430070, China; 202120390@mail.sdu.edu.cn (X.T.); wangyuan1034@126.com (Y.W.); zrl605290594@163.com (R.Z.); hanwenyuan@mail.hzau.edu.cn (W.H.); nanp@mail.hzau.edu.cn (N.P.); fa-lyx@163.com (Y.X.L.); 3 Department of Biology, University of Copenhagen, Ole Maaløes Vej 5, 2200 Copenhagen, Denmark; jinzhong.lin@bio.ku.dk; 4 CRISPR and Archaea Biology Research Center, State Key Laboratory of Microbial Technology, Shandong University, 72 Binhai Road, Qingdao 266237, China; wuchenweiwcw@163.com (C.W.); 18339193239@163.com (X.W.); ppz1997@126.com (P.Z.); 18232575953@163.com (X.B.); lqszwsw@163.com (Z.Y.)

**Keywords:** CRISPR-Cas system, target RNA cleavage, Cmr4, spatiotemporal regulation of Cmr systems, autoimmunity, RNA-activated DNase, cOA synthesis, Sulfolobales

## Abstract

Type III CRISPR-Cas systems show the target (tg)RNA-activated indiscriminate DNA cleavage and synthesis of oligoadenylates (cOA) and a secondary signal that activates downstream nuclease effectors to exert indiscriminate RNA/DNA cleavage, and both activities are regulated in a spatiotemporal fashion. In III-B Cmr systems, cognate tgRNAs activate the two Cmr2-based activities, which are then inactivated via tgRNA cleavage by Cmr4, but how Cmr4 nuclease regulates the Cmr immunization remains to be experimentally characterized. Here, we conducted mutagenesis of Cmr4 conserved amino acids in *Saccharolobus islandicus*, and this revealed that Cmr4α RNase-dead (dCmr4α) mutation yields cell dormancy/death. We also found that plasmid-borne expression of dCmr4α in the wild-type strain strongly reduced plasmid transformation efficiency, and deletion of CRISPR arrays in the host genome reversed the dCmr4α inhibition. Expression of dCmr4α also strongly inhibited plasmid transformation with Cmr2α^HD^ and Cmr2α^Palm^ mutants, but the inhibition was diminished in Cmr2α^HD,Palm^. Since dCmr4α-containing effectors lack spatiotemporal regulation, this allows an everlasting interaction between crRNA and cellular RNAs to occur. As a result, some cellular RNAs, which are not effective in mediating immunity due to the presence of spatiotemporal regulation, trigger autoimmunity of the Cmr-α system in the *S. islandicus* cells expressing dCmr4α. Together, these results pinpoint the crucial importance of tgRNA cleavage in autoimmunity avoidance and in the regulation of immunization of type III systems.

## 1. Introduction

CRISPR-Cas (clustered regularly interspaced short palindromic repeats, CRISPR-associated) systems provide adaptive immunity in archaea and bacteria. The immune system defends against invasive genetic elements in three steps. Upon the first invasion of a virus, the system recognizes a DNA sequence from its genome, and cleaves and inserts the DNA sequence into a CRISPR array as the first new spacer. Then, the system produces mature CRISPR RNA (crRNA) from transcripts of the CRISPR array, and in the interference stage, the crRNA and Cas proteins form ribonucleoprotein (RNP) complexes that restrict the re-occurring virus, specifically in an RNA-guided fashion [1,2,3,4,5,6,7,8,9]. CRISPR-Cas systems fall into two classes and at least six different types. Type III CRISPR-Cas systems belong to the Class 1 group, since they possess an RNP with multiple Cas proteins, and represent the most complex type. 

Currently, six type III subtypes (A to F) are known, of which a few III-B (also called Cmr) and III-A (belonging to Csm) systems [10,11] have been characterized. These immune systems exhibit three distinct activities [12,13,14]: (a) tgRNA cleavage occurring with a 6-nt periodicity [15,16,17,18,19], which is mediated by a conserved Asp residue in the active center of Cmr4 or Csm3 [17,20,21], the large backbone subunit in each subtype; (b) the tgRNA-activated DNA cleavage mediated by the HD domain of the Cas10 subunit (Cmr2 or Csm1) [22,23,24,25,26]; and (c) the synthesis of cyclic oligoadenylates (cOAs) upon binding to tgRNA by the Cas10 Palm domains, and cOAs function as a second messenger to activate the nuclease activity of CARF domain proteins (the cOA nuclease effectors), including Csm6/Csx1/Can1/NucC/Card1 to induce cell dormancy to curb the virus infection [27,28,29,30,31,32,33,34,35,36]. 

All immune activities of CRISPR-Cas systems must be strictly controlled to avoid autoimmunity (self-targeting), and self-targeting avoidance is particularly important for type III immune systems since their DNase and cOA effector nucleases are sequence-independent [22,23,24,25,27,28,37,38,39,40,41]. If not controlled properly, both interference activities can yield cell dormancy or cell death in vivo [32,37]. 

Currently, self-targeting avoidance in type III CRISPR systems involves three distinctive controls. The first mechanism provides discrimination at the RNA level. While mismatches between the 5′-handle of crRNA and the 3′-flanking region of tgRNA activate the type III effector for DNA cleavage and cOA generation, their complementarity suppresses both activities [22,23,24,25,27,28,42]. The second mechanism is tgRNA cleavage by the backbone subunits Cmr4 and Csm3, which inactivate the type III effectors and only allow the immunization to occur within a brief time period [22,23,24,27,28]. The last control is the presence of ring nucleases that degrade cOA signal molecules to terminate the CRISPR signaling pathway [43,44,45,46,47,48,49,50,51,52,53]. Note that the second mechanism, i.e., the spatiotemporal control of the type III immunity, was suggested by biochemical experiments but has not been characterized genetically. 

*Saccharolobus islandicus* (formerly *Sulfolobus islandicus*) REY15A has been used as a model crenarchaeon for investigation of molecular mechanisms of CRISPR-Cas systems [54]. The organism codes for three different immunities, a type I-A and two type III-B (Cmr-α and Cmr-β), all of which are active in antiviral defense [55,56,57]. The *S. islandicus* Cmr-α has been characterized in detail, including the discovery of the transcription-dependent DNA interference and DNA and RNA dual interference for type III CRISPR-Cas systems [58,59], the demonstration of RNA-activated ssDNA cleavage and synthesis of cyclic tetraadenylates (cA4) by this IIIB CRISPR-Cas [25,30] and the activation of Csx1 by the cA4 secondary signal [41,60]. In addition, functions of the Cmr1α and Cmr3α subunits have been characterized in vivo and in vitro [61,62,63]. Genetic study of the *cmr4* genes in the two III-B immune systems revealed that the Cmr4β^D31A^ mutation was readily constructed [55], but attempts to generate the corresponding Cmr4α^D27A^ mutant failed consistently. A series of *cmr4α* mutants were constructed that carried substitution mutation(s) in the conserved amino acids, and then characterized. We found that inactivation of the Cmr4α active site yields self-targeting that is lethal to the crenarchaeal cells, and the lethal toxicity can be facilitated by either the Cmr-α DNase or cyclase. 

## 2. Results

### 2.1. Inactivation of the Cmr4α RNase Induces Cell Dormancy or Cell Death to S. islandicus

In the *S. islandicus* Cmr-α system, Cmr4α is the large backbone subunit implicated in tgRNA cleavage [25]. In the *P. furiosus* Cmr system, Cmr4 functions in the spatiotemporal regulation of the DNase and cyclase of the Cmr effector complexes [64]. Here, we genetically characterized the *S. islandicus* Cmr4 regulation of the type III CRISPR immunity. First, attempts were made to construct strains carrying alanine substitution for a selected set of conserved amino acids of Cmr4α. These included H16, D27, K46/K50, W197, E199/Y201, G244/G245/G250/G252 (denoted as 4G) and K251 (Supplementary Figure S1), among which D27 is the predicted active site for RNA cleavage in the Cmr-α system [19,20,21,65]. Since *S. islandicus* REY15A encodes two III-B CRISPR-Cas systems, Cmr-α and Cmr-β, both of which are active in mediating antiviral defense [12,55,56], *S. islandicus* strains lacking all Cmr-β genes (ΔCmr-β, Supplementary Table S1) were used as the genetic host for the functional analysis of Cmr4, to eliminate any possible interference from the Cmr-β system. 

Genome editing plasmids (pGE-4αH16A, -D26A, -K46A/K50A, -W197A, -E199A/Y201A, -4GA and -K251A) were designed and constructed for each of these Cmr4α substitutions (Supplementary Table S1). Each pGE plasmid was then introduced into ΔCmr-β cells by electroporation in order to generate these Cmr4α mutants by following an endogenous CRISPR gene-editing procedure (Supplementary Figure S2) [66]. We found that all except pGE-4α-D27A gave a high transformation efficiency, whereas the latter exhibited a ca. 50-fold lower rate of transformation. Genotypes of transformants were checked by PCR amplification of the *cmr4α* gene and sequencing of the resulting PCR products. We found that designed Cmr4α mutants were obtained for all pGE plasmids showing a high transformation rate, including for H16A, D27A, K46A/K50A, W197A, E199A/Y201A, 4GA and K251A substitutions; for pGE-4α-D27A, the rates of transformation with the plasmid were low, and colonies of all tested transformants carried the wild-type *cmr4α* gene. This indicated that the conserved D27 could function as the active site of the Cmr4α nuclease, as shown for the *P. furiosus* Cmr4 [20], and furthermore, inactivation of the tgRNA cleavage activity could induce cell dormancy or cell death in the crenarchaeal cells. 

### 2.2. The dCmr4α-Induced Cell Death Is Strictly Dependent on the Activities of Cmr2α

**Figure 1:**
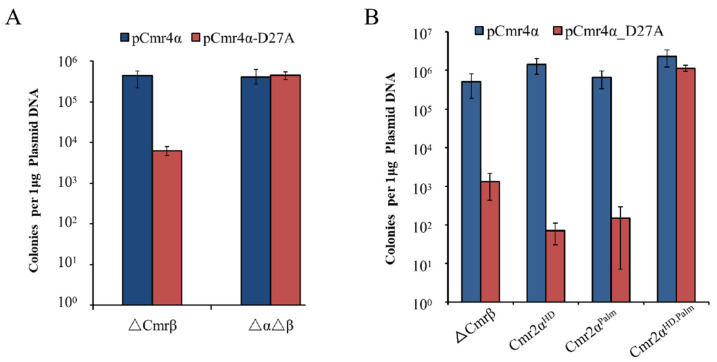
The dCmr4α-induced cell death is dependent on the activities of Cmr2α. (**A**) Deletion of the *cmr-α* module abolished the Cmr4α-D27A toxicity. pCmr4α and pCmr4α-D27A were transformed into ∆βE233S1 and ∆α∆βE233S1, respectively, and the transformation efficiency was calculated. (**B**) Cmr4α-D27A toxicity in ∆βE233 and the *cmr2α* mutant strains. pCmr4α and pCmr4α-D27A were transformed into ΔβE233(WT) and the *cmr2α* mutants, respectively, and the transformation efficiency was calculated.

To test whether the observed Cmr4α-D27A toxicity could be attributed to the mutated protein itself, we deleted the Cmr-α locus in the genetic background of ΔCmr-β, yielding the ΔαΔβ strain. Then, the mutant and the original strain (ΔCmr-β) were transformed with pCmr4α or pCmr4α-D27A (Supplementary Table S2), where the former expresses the wild-type Cmr4α, and the latter, a predicted nuclease-dead derivative, dCmr4α (Cmr4α-D27A). We found that transformation of ΔαΔβ with both plasmids gave very similar efficiencies of transformation, indicating that the Cmr4α-D27A protein is not itself toxic. However, transformation of ΔCmr-β with pCmr4α-D27A showed about 100-fold lower efficiency of transformation relative to pCmr4α (Figure 1A). Since the only difference between the two strains is that ΔCmr-β has retained the Cmr-α module, these results indicated that the plasmid-borne expression of dCmr4α in *S. islandicus* induced cell lethality in archaeal cells carrying an active Cmr-α system. This phenomenon was termed dCmr4α-induced cell dormancy or cell death (dCmr4-ICD). 

Previous investigations into the two Cmr2-based activities using an interference plasmid assay (Supplementary Figure S3) in *S. islandicus* revealed that only the Palm mutation impaired the Cmr-α immunization, whereas mutation of the HD domain impaired the Cmr-α ssDNase but did not influence the transformation rate of interference plasmids [25,30]. This raised the question of whether and how the Cmr-α ssDNase contributes to the Cmr-α immunization. Here, we attempted to develop an in vivo assay based on dCmr4-ICD to investigate the immune response of the Cmr-α DNase and cyclase. 

Three *cmr2α* mutants were constructed with *S. islandicus* Δβ, giving Cmr2α^HD,^ Cmr2α^Palm^ and Cmr2α^HD,Palm^, which individually carry an inactive HD domain (H14A, D15A) and an inactive Palm2 domain (D667A, D668A), as well as a combination of both mutated domains. All three mutants were then transformed with pCmr4α-D27A. We found that transformation efficiency with pCmr4α−Δ27A was greatly reduced in the wild-type strain and two Cmr2α single-domain mutants (100–1000 folds), whereas transformation rates of the mutant carrying double domain mutations were very similar with both expression plasmids (Figure 1B). These results indicated that in the presence of dCmr4α, the Cmr-α DNase activity can also mediate immune responses in the archaeal cells. 

In addition, the successful transformation of Cmr2α^HD,Palm^ with pGE-4α-D27A rendered it possible to test whether the mutated Cmr proteins could be assembled into a ribonucleoprotein effector complex. The triple mutant was employed for purification of the Cmr-α effector complex, and a large protein complex was obtained. SDS-PAGE analysis of the purified effector complexes revealed that the mutated Cmr-α retained its integrity, since the stoichiometry of its subunits was comparable to that of the wild type (Supplementary Figure S4A). This RNP was designated as Cmr-α-2α^HD,Palm^4α^D27A^ and tested for tgRNA cleavage, RNA-activated DNA cleavage and cOA synthesis. As would be expected, the mutated effector lacked all three activities (Supplementary Figure S4B–D). Hence, we concluded that the dCmr4α toxicity relies on the Cmr2α activities in the effector complex, and each Cmr2α activity, i.e., either the RNA-activated ssDNase or the RNA-activated cOA synthesis, is capable of mediating the dCmr4-ICD in *S. islandicus* cells. In addition, this also experimentally demonstrated that D27 is the active site of the Cmr4α nuclease, and the backbone RNA cleavage activity is very important for the regulation of the Cmr-α system immunity in *S. islandicus*.

### 2.3. The Cmr2α-Based Activities Are Not Equally Efficient in Mediating the Antiviral Defense 

It is noteworthy that, while the interference plasmid assay could not reveal the immune responses of the Cmr2α DNase in *S. islandicus* [25], the transformation of pCmr4α-D27A could do so. An apparent difference between the two experimental setups was that upon activation, the Cmr-α_d4α effector complexes formed with dCmr4α would be constantly active, whereas the wild-type Cmr-α is only active in a brief time window due to the spatiotemporal regulation. In this scenario, it would be interesting to test whether the immune responses of the Cmr-α DNase could be revealed by elevating the CTR levels, to enlarge the time window of the active Cmr-α DNase. 

**Figure 2:**
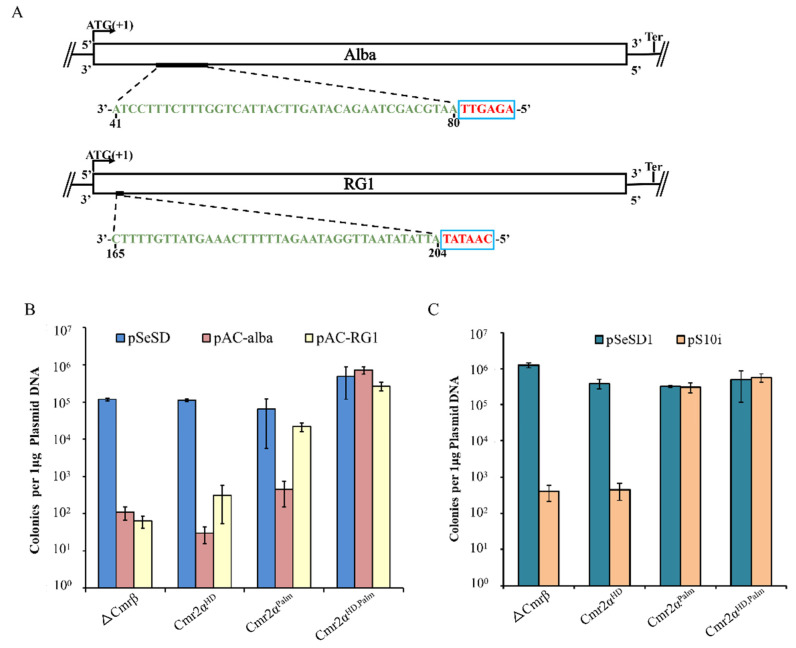
The Cmr2α-based activities are not equally efficient in mediating the antiviral defense. Self-targeting activity in the *cmr2α* mutant strains. (**A**) Outline of designed target sites for testing Cmr-α immunity in *S. islandicus*. (**B**) The control plasmid pSeSD1 and the self-targeting plasmids pAC-alba and pAC-RG1 were transformed into ∆βE233(WT) and the *cmr2α* mutant strains, respectively, and the transformation efficiency was calculated. (**C**) The control plasmid pSeSD1 and the self-targeting plasmid pS10i were transformed into ∆βE233(WT) and the *cmr2α* mutant strains, respectively, and the transformation efficiency was calculated. *Alba* encodes a crenarchaeal chromatin protein; *RG1* codes for a reverse gyrase topoisomerase.

We chose to design self-targeting assays to study this question. SiRe_1125 and SiRe_1581 were selected as target genes (Figure 2A) to reveal the influence of tgRNA levels on the Cmr-α immunization. SiRe_1125 codes for Alba, a chromatin protein [67] that also functions as an RNA chaperon [68], and SiRe_1581 encodes a reverse gyrase (RG1) that protects damaged DNA sites in vivo [69]. In previous studies, RNA seq data and qPCR analysis showed that the two genes are expressed at the level of >300-fold difference [70,71]. Cmr-α-targeting protospacers were identified in the template strand of the coding region of each gene, with which oligos were designed for the generation of spacer fragments. Insertion of the spacer fragments into pSe-Rp yielded pAC-alba and pAC-RG1 individually (Supplementary Table S2). 

These plasmids, pAC-alba, pAC-topR1 and the expression vector pSeSD, were then introduced into ΔCmr-β, Cmr2α^HD^, Cmr2α^Palm^ and Cmr2α^HD,Palm^ individually by electroporation. Their transformation efficiency was calculated, and the results are shown in Figure 2B. We found that: (a) transformation of ΔCmr-β with the two pAC plasmids yielded very similar transformation rates, which were ca. 1000-fold lower than the transformation with pSeSD, a reference plasmid, and these results indicated that the self-targeting activity by the wild-type Cmr-α effector is lethal to the archaeal cells at both expression levels; (b) in the Cmr2α^HD^ mutant that retained the cA4 signaling pathway, the mutated immune system (Cmr-α-2α^HD^) still retained the Cmr-α self-targeting activity; (c) the Palm mutation rendered the system (Cmr-α-2α^Palm^) inefficient in mediating self-targeting with the tgRNA at the level of the reverse gyrase 1 (RG1) gene expression; and (d) inactivation of both Cmr2 activities completely abrogated the self-targeting activity. In comparison, evaluation of the Cmr-α immunity in the three Cmr2α mutants using an interference plasmid assay (Supplementary Figure S3B) showed that only the Palm domain alone, i.e., Cmr2α^HD^, reduced the plasmid transformation rate (Figure 2C), as previously reported [25]. Taken together, we conclude that, upon activation, both Cmr2α domains are capable of exerting immunization. Further, the CRISPR signaling pathway provides a more powerful immune mechanism, relative to that from the HD domain-based ssDNA cleavage, in the *S. islandicus* Cmr-α system.

### 2.4. The dCmr4 Self-Targeting Is Dependent on the Presence of Genomic CRISPR Loci

Nevertheless, how the dCmr4α mutation could induce dCmr4-ICD in *S. islandicus* in the absence of any identifiable tgRNA remained an intriguing question. Previously, it was shown that type III systems can tolerate a number of mismatches between crRNAs and their targets in mediating immune responses [72]. We reasoned that the magnitude of this tolerance would be greatly amplified in the dCmr4 mutant, which would turn ineffective self-targeting events in the wild-type Cmr-α strain into effective ones in the dCmr4α mutation. There are two CRISPR loci in *S. islandicus* producing > 200 different species of crRNA, and these crRNAs could show a limited sequence homology to certain species of cellular RNAs. In this scenario, the occurrence of dCmr4-ICD in this archaeon would require the presence of the genomic CRISPR arrays.

The assumption was then tested by construction of the ΔCRISPR mutant lacking any spacer and transformation of the mutant strain with pCmr4α and pCmr4α-D27A. We found that dCmr4α showed little influence on plasmid transformation efficiency (pCmr4α vs. pCmr4α-D27A), indicating that dCmr4-ICD relies on the production of crRNAs and, presumably, their subsequent interactions with cellular RNAs.

### 2.5. dCmr4α-Induced Self-Targeting Is Still Subject to the NTR Protection

**Figure 3:**
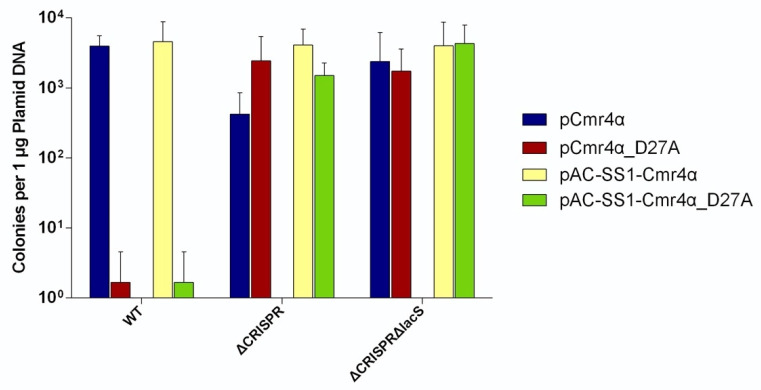
Contribution of CRISPR array and target sequence to the Cmr4α-D27A toxicity. Transformation efficiency was determined for pCmr4α and pCmr4α-D27A plasmids individually with three different host strains: ∆βE233(WT), ∆CRISPR∆β and ∆CRISPRΔlacS∆β(∆array).

In all known type III CRISPR-Cas systems, NTR protection provides an important mechanism for self-targeting avoidance, as in all known type III immune systems. The occurrence of dCmr4-ICD in *S. islandicus* prompted us to test whether NTR could still silence the immunization of the dCmr4α-containing RNP. For this purpose, a new strain and a new set of plasmids were constructed, including the ΔCRISPRΔlacS strain, which was constructed by removing the *lacS* gene from ΔCRISPR, and two plasmids, pAC-SS1-Cmr4α and pAC-SS1-Cmr4α-D27A, which carry an artificial CRISPR array with an SS1 spacer in addition to the *cmr4α* gene (Supplementary Tables S1 and S2). In previous studies, the 5′-GAAAG-3′ sequence in the *lacS* transcript was found to effectively prevent self-targeting in this model, allowing only the tgRNA cleavage to occur [58,62]. These host strains were then transformed with pAC-SS1-Cmr4α and pAC-SS1-Cmr4α-D27A to test the NTR protection of dCmr4-ICD. We found that, when plasmid-borne expression yielded both dCmr4α and SS1 crRNA (the latter matching the corresponding NTR in the *lacS* mRNA), the prolonged interaction between SS1 crRNA and the NTR failed to induce dCmr4-ICD (Figure 3). This indicated that the activation of this III-B effector strictly requires the mismatches between the 5′-repat handle of crRNAs and the 3′-flanking motif of the tgRNAs, reinforcing the fact that the basic principle of tgRNA activation always operates, even for effectors with an everlasting immunity. 

### 2.6. Function of Cmr4α Conserved Amino Acids in the Cmr-α Immune System 

To investigate the functional roles of the conserved amino acids in Cmr4α, all constructed mutants, including H16A, K46A/K50A, W197A, E199A/Y201A, 4GA and K251A substitutions, were analyzed by two genetic assays developed for *S. islandicus* (Supplementary Figure S3): the mini-CRISPR and reporter-gene-based RNA interference assay and the interference plasmid assay. 

**Figure 4:**
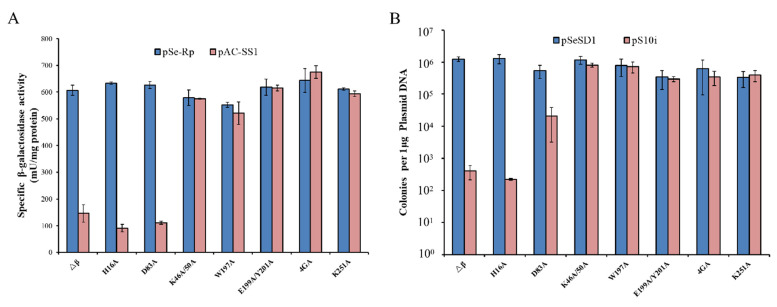
Function of Cmr4α conserved amino acids in the Cmr-α immune system. (**A**) In vivo RNA interference activity in the *cmr4α* mutant strains determined by artificial mini-CRISPR-based reporter gene assay. The chromosomal *lacS* gene was used as the reporter gene. pSe-Rp: reference plasmid; pAC-SS1: an artificial mini-CRISPR plasmid carrying S1 spacer of the *lacS* gene. (**B**) Invading plasmid silencing activity of the *cmr4α* mutant strains. pSeSD1: the reference plasmid; pS10i: an invading plasmid carrying a target sequence of spacer 10 in CRISPR locus 2 in *S. islandicus* REY15A; Δβ: the wild-type strain; other strains: mutants carrying the specified point mutation of Cmr4α; 4GA: G244AG245AG250AG252A substitutions.

The RNA interference assay was conducted as follows. The *cmr4α* mutants were transformed with two plasmids: (1) a mini-CRISPR plasmid (pAC-SS1) expressing a crRNA that is complementary to a fragment of *lacS* mRNA and (2) the corresponding reference plasmid pSe-Rp. The resulting transformants were grown in SCVy medium, and the cell mass was collected by centrifugation and used for the preparation of cell-free extracts. The β-glycosidase activity in each transformant was then determined using the ONPG method. The rationale is that crRNAs produced from pAC-SS1 guide the wild-type or mutated Cmr-α effector to specifically identify the target in the *lacS* mRNA, mediating RNA degradation. Therefore, the β-glycosidase activity is reversely correlated with the RNA interference activity of the Cmr-α complex in *S. islandicus* cells. We found that, in the presence of pAC-SS1, strains expressing the wild type as well as the H16A and D83A substitutions of Cmr4α retained about 20% of the reporter gene activity, relative to the transformants of pSe-Rp, the reference plasmid. However, five *cmr4α* mutants (i.e., K46A/K50A, W197A, E199A/Y201A, 4GA and K251A) showed a β-glycosidase activity comparable to that of their pSe-Rp transformants (Figure 4A). These results indicated that these Cmr4α mutations have essentially abolished the RNA interference of the Cmr-α system. 

For the interference plasmid assay, these *cmr4α* mutant strains were transformed with two plasmids: pSeSD1, a reference plasmid and pS10i, the interference plasmid that contains a transcribed target complementary to endogenous spacer 10 of CRISPR locus 2 and can be silenced by Cmr-α interference, thereby reducing the transformation rate [59]. The resulting data showed that these mutants fell into three groups (Figure 4B). (a) The first group included *cmr4α* mutants of K46A/K50A, W197A, E199A/Y201A, 4GA and K251A, which showed similar transformation efficiency with the reference plasmid pSeSD1 and the interference plasmid pS10i, indicative of deficiency in plasmid silencing. (b) The Δβ strain and the Cmr4α^H16A^ mutant showed about 1000-fold lower efficiency in plasmid transformation with pS10i, relative to pSeSD1, suggesting that the H16A substitution does not influence the plasmid silencing capability. (c) The Cmr4α^D83A^ mutant showed a 10-fold difference in plasmid transformation with pSeSD vs. pS10i, indicating that the D83A substitution impairs DNA interference in the Cmr-α system.

To investigate whether the loss of RNA and DNA interference could be a result of the lack of effector complex assembly capability in the Cmr4α mutants, we attempted to purify Cmr-α complexes from all constructed Cmr4α mutant strains using His-tag-Cmr6α copurification, as previously described [25,62]. Native Cmr-α complexes were obtained from the Cmr4α^H16A^ and Cmr4α^D83A^ substitution strains (Supplementary Figure S2) but not from any of other *cmr4α* mutants, indicating that K46/K50, W197, E199/Y201, G244/G245/G250/G252 and K251 of Cmr4α have an essential role in assembly of the Cmr-α effector complex and/or in the maintenance of its integrity. 

**Figure 5:**
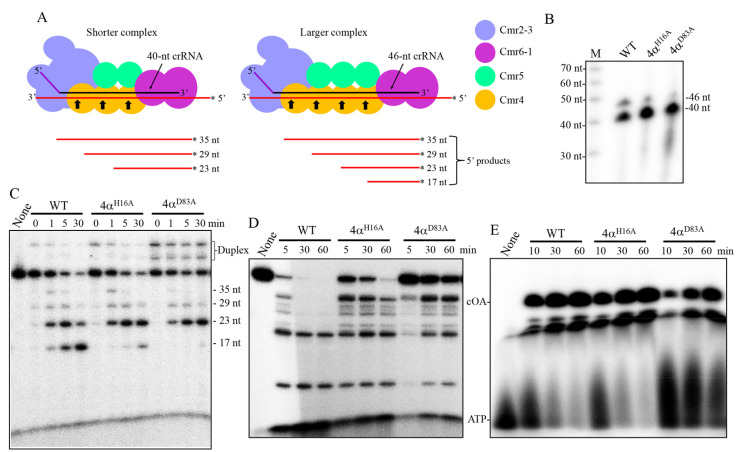
crRNA distribution pattern and activities of Cmr-α_4α^H16A^ and Cmr-α_4α^D83A^. (**A**) Schematic map of the shorter (carrying a 40-nt crRNA and three Cmr4 subunits) and the longer (carrying a 46-nt crRNA and four Cmr4 subunits) Cmr-α complexes and their RNA cleavage products. Asterisk symbol at each RNA end indicates radio-labeling. (**B**) crRNA distributions of WT (wild-type Cmr-α), 4α^H16A^ and 4α^D83A^ represent the Cmr-α effectors carrying the indicated Cmr4α mutation (i.e., Cmr-α_4α^H16A^ and Cmr-α_4α^D83A^). Activities of the three Cmr-α complexes: tgRNA cleavage (**C**), ssDNA cleavage (**D**) and cOA synthesis (**E**). Cleavage assay was conducted for the time periods indicated in the figures; smaller fragments in panel C and D panels represent cleavage products. The sizes of RNA cleavage products in 6-nt periodicity are indicated. None: substrate only, no Cmr complex was added; duplex: duplex of crRNA and substrate; cOA: cyclic oligoadenylates.

The mutated Cmr-α effector complexes carrying Cmr4α^H16A^ or Cmr4α^D83A^ (named Cmr-α_4α^H16A^ and Cmr-α_4α^D83A^) were analyzed for their crRNA content. The RNA component was extracted from the purified effector complexes by RNA extraction, radio-labeled and separated by denaturing PAGE, and this revealed that they also carried crRNAs of two different sizes, as in the wild-type Cmr-α. Nevertheless, the relative content of the two crRNA species was different. While the wild-type Cmr-α possessed ca. 72% of 40-nt crRNA versus ca. 28% of 46-nt crRNA, the crRNA composition in Cmr-α_4α^H16A^ and Cmr-α_4α^D83A^ was 95% vs. 5% and 98% vs. 2%, respectively (Figure 5B). Since the 46-nt and 40-nt crRNA is bound by two distinct Cmr-α effector complexes containing four and three copies of Cmr4α, respectively (Figure 5A) [73], these data suggested that alanine substitution of H16 and D83 impaired the incorporation of a fourth subunit of Cmr4α into the effector complex. Indeed, an RNA cleavage assay showed that both mutated complexes efficiently cleaved tgRNA but generated much less 17-nt product than the wild type (Figure 5C). Analysis of the RNA-activated DNA cleavage activity of the mutated complexes revealed that their RNA-activated ssDNase was strongly impaired (Figure 5D), but the two mutations showed different effects on the cOA synthesis, since similar amounts of cOA were synthesized by Cmr-α_4α^H16A^ and the wild-type effector, whereas D83A strongly impaired the cOA production (Figure 5E). These results indicated that H16 and D83 play different roles in the activation of the Cmr-α immunity by a cognate tgRNA. 

## 3. Discussion

It is widely accepted that Csm3/Cmr4 proteins function in the spatiotemporal regulation of the RNA-activated ssDNase and cOA synthesis hosted by Cas10 (Csm1 or Cmr2). However, genetic analysis of several *csm3* genes indicated that bacterial cells are viable upon the inactivation of the backbone nuclease, including the *S. epidermidis* Csm [39], the *S. thermophilus* Csm [17] and the *Lactobacillus delbrueckii* subsp. *bulgaricus* Csm [26]. Here, we show that generation of a nuclease-dead Cmr4α (dCmr4α) mutant by amino acid substitution at the active site induces cell dormancy or cell death in *S. islandicus*, which is in good agreement with the model. We have further revealed that the dCmr4α-induced cell death is CRISPR-array-dependent, and the autoimmunity can occur in the absence of any known tgRNA in *S. islandicus*. This paradoxical phenomenon can be explained by the possibility that Cmr-α_4α^D27A^ could have broadened its tgRNA range compared to the wild-type effector complex, probably by tolerating more mismatches between crRNA and tgRNA. In this scenario, cellular RNAs, which could interact with crRNAs due to their sequence complementarity but were incapable of triggering self-targeting in the wild-type Cmr-α system, would then elicit the immunization in the presence of dCmr4α. This could account for the robust self-targeting observed in the Cmr4α^D27A^ mutagenesis and dCmr4α expression experiments in this study. The finding that deletion of the chromosomal CRISPR array abrogates dCmr4-ICD events further supports the assumption.

When tgRNA cleavage activity was discovered in 2009, it was proposed that the activity could silence targeted viral genes to block the viral life cycle [15]. Indeed, the backbone cleavage can reduce the mRNA level of target genes in vivo and result in loss of function of a number of targeted genes in Sulfolobales [7,58,69,74,75,76]. In addition, the *S. thermophilus* Csm can prevent infection of *E. coli* cells by an ssRNA virus [17]. However, characterization of the *Staphylococcus epidermidis* Csm system revealed that *csm3* is not required for anti-plasmid immunity in *Staphylococcus aureus* [77]. Subsequently, type III CRISPR-Cas systems were found to mediate tgRNA-activated indiscriminate DNA cleavage and cOA synthesis activity, whereas the tgRNA cleavage and release function in the deactivation of the Cas10 activities to avoid self-immunity [22,23,24,25,27,28]. In this model, Csm3 and Cmr4 proteins function as a terminator to the tgRNA-activated indiscriminate DNA cleavage and cOA synthesis activity. If the active site conserved in Cmr4 and Csm3 proteins constitutes the only RNase activity controlling tgRNA turnover in the ternary effector complexes, inactivation by substitution mutation would yield an everlasting immune activity, yielding robust self-targeting. Indeed, this is exactly what we have observed for the *S. islandicus* Cmr-α system in this study, and these data provide strong genetic evidence to support the model of the spatiotemporal regulation of type III immune machineries.

In the current literature, it appears the extent of diversification in the control of Cas10 activities by the backbone nuclease Cmr4/Csm3 is large. First, three bacterial Csm systems have been characterized for the tolerance of a nuclease-dead Csm3 (dCsm3) mutation, and the mutant has been obtained for all three systems [24,26,39]. Expression of the dCsm3 mutant of the *S. epidermidis* Csm system in *S. aureus* still yields viable cells in the presence of a cognate tgRNA [39,77]. In addition, mutated Csm_dCms3 effector complexes have been purified, and biochemical characterization showed they all lack backbone RNA cleavage activity and show enhanced RNA-activated ssDNA cleavage activity [24,26,77]. These results are consistent with genetic analysis of the ΔCmr4β mutation of the *S. islandicus* Cmr-β system [55] but are in contrast to the *P. furiosus* Cmr [64] and *S. islandicus* Cmr-α systems (this work). In the two latter systems, ΔCmr4 mutation cannot be obtained when the archaeal cells carry an active type III immune system. Interestingly, in the *S. islandicus* Cmr-β system, its Cas10 protein, Cmr2β, possesses RNA-activated RNase that cleaves cellular RNAs at the UA site [55], as for the *S. solfataricus* Cmr system [19,78], and this RNase activity could then render the *S. islandicus* ΔCmr4β cells viable, since the activity can eventually destroy all tgRNAs. However, how the Cas10 activities in other type III immune systems, e.g., the Csm-dCsm3 effector complexes, are terminated remains elusive. Nevertheless, the *Streptococcus thermophilus* Csm effector exhibits significant disparity between the backbone cleavage and Cas10 deactivation [24,27], and this is in contrast to the prompt disassociation of the cleavage product after backbone cleavage and quick deactivation of the cOA synthesis observed for a type III-D Csm effector from *S. solfataricus* [29]. In addition, prompt disassociation of the cleavage product has also been observed for DNA cleavage by the *S. islandicus* Cmr-α effector [25]. Hence, type III-A systems could have evolved new mechanisms for Cas10 deactivation during evolution. 

Our genetic analysis has revealed several conserved motifs in SisCmr4α that are essential for the formation of the Cmr-α complex, including K46/K50, W197, E199/Y201, G244/G245/G250/G252 (4G) and K251. Alanine substitution of these amino acids abolished both in vivo RNA interference activity and plasmid interference activity, which is consistent with the failure of the attempts to purify the corresponding native effector complexes in these *cmr4α* mutants. These results are in good agreement with the predicted functions of these conserved amino acid residues, as: (a) K46 and K50 are predicted to bind crRNA by interaction with the backbone phosphates [79]; (b) W197, E199, and Y201 are located in a thumb-like structure, which intercalates between duplexed crRNA and tgRNA and places the scissile site of tgRNA close to the active site [73,79]; (c) the C-terminal G-rich loop has been implicated in the formation of an inner surface of the Cmr4 filament, which is supposed to bind to crRNA [80]; and (d) the conserved basic residue K251 is located in the G-rich loop, which forms a salt bridge with the D83 residue of the adjacent Cmr4 subunit as predicted by the structural analysis of *Pyrococcus furiosus* Cmr4 (the corresponding residues are K279 and D86 in PfuCmr4) [21]. 

Taken together, we have genetically demonstrated that both Cmr DNase and cyclase activities exert immune responses, and tgRNA cleavage by Cmr4 functions in preventing autoimmunity of III-B CRISPR-Cas systems. We have also revealed that NTRs, which could be produced from transcription of the opposite strand of the CRISPR array [81], are very powerful in turning off the immune responses of III-B CRISPR-Cas systems, providing an additional level of autoimmunity avoidance.

## 4. Materials and Methods

### 4.1. Strains, Growth Conditions and Transformation of Saccharolobus 

All *Saccharolobus* strains were derived from the *S. islandicus* E233 [82], a spontaneous *pyrEF* deletion mutant isolated from the original isolate *S. islandicus* REY15A [83,84]. The *cmr4α* and *cmr2α* mutant strains (Supplementary Table S1) were generated by the CRISPR-based genome-editing procedure, [66] using ΔCmr-β as the host (Supplementary Table S1). *S. islandicus* strains were grown at 78 °C in SCV medium (basic salts plus 0.2% sucrose, 0.2% casamino acids and 1% vitamin solution) or SCVy (SCV plus 0.0025% yeast extract), with uracil supplemented to 20 μg/mL if required, as described previously [82]. *Saccharolobus* competent cells were prepared and transformed by electroporation, as previously described [82].

### 4.2. Construction of Plasmids

The genome editing plasmids (Supplementary Table S2) were constructed as described previously [66]. Taking the pGE-4α-D27A construction as an example: the spacer fragment was generated by annealing 4α-D27A-SpF and 4α-D27A-SpR (Supplementary Table S3), and insertion of the spacer fragment into BspMI-digested pSe-Rp gave pAC-4α-D27A; the donor DNA containing the 4α-D27A mutant allele was obtained by splicing and overlap extension PCR (SOE PCR [85]) using primers 4α-D27A-SOER and 4α-D27A-SOEF as two overlapping primers and 4α-D27A-SalIF and 4α-D27A-NotIR as two flanking primers (Supplementary Table S3); and insertion of the donor DNA into pAC-4α-D27A yielded pGE-4α-D27A.

Cmr4α overexpression plasmid pCmr4α was constructed as follows. The *cmr4α* gene was amplified with the primers Cmr4α-fwd and Cmr4α-rev (Supplementary Table S3) and inserted into pSeSD1 at the NdeI and SalI sites. Construction of pCmr4α_D27A was conducted by generation of the *cmr4α* mutant gene by SOE-PCR, with the primers 4α-D27A-SOER and 4α-D27A-SOEF as overlapping primers and Cmr4α-fwd and Cmr4α-rev as flanking primers (Supplementary Table S3). The resulting gene fragment was then inserted into the NdeI and SalI sites of the expression vector to give pCmr4α_D27A. 

CRISPR plasmids pAC-Alba and pAC-RG1 (Supplementary Table S2) were constructed as described for pAC-SS1 [58]. The spacer fragment was generated by annealing of Si_1125-up and Si_1125-dw (for the *alba* gene coding for the chromatin protein Alba) and Si_1581-up and Si_1581-dw (for the *RG1* gene encoding a reverse gyrase enzyme) individually (Supplementary Table S3), and the resulting mini-CRISPR arrays were then individually inserted into the BspMI-digested pSe-Rp to yield the CRISPR plasmids.

All the oligonucleotides were synthesized by Tsingke (Wuhan, China) and the sequences of all plasmid constructs were verified by DNA sequencing (Tsingke, Wuhan, China).

### 4.3. Determination of β-Glycosidase Activity

*S. islandicus* strains were grown in SCVy medium to an A_600_ (absorbance at 600 nm) of about 0.3. The cell mass was then collected by centrifugation for each culture for which cellular extracts were prepared. Protein content in the cellular extracts was determined using BCA Protein Assay Reagent (Thermo Scientific), whereas the β-glycosidase activity was determined using the ONPG (*ρ*-nitrophenyl-β-D-galactopyranoside) method, as described previously [86].

### 4.4. Purification of Cmr-α Ribonucleoprotein Complex

*S. islandicus* strains (ΔCmr-β and *cmr4α* mutants) were transformed with pAC-cmr6α-10His plasmid, giving transformants for Cmr-α complex purification [25]. The transformants were grown in SCVy medium, and a total of 10–12 L of culture was prepared for each strain. When the A_600_ of the cultures reached 0.7–0.8, cells were collected by centrifugation. A cell pellet was re-suspended in Buffer A (20 mM HEPES pH 7.5, 20 mM imidazole, 250 mM NaCl) and disrupted using a French press, followed by centrifuging at 12000 rpm for 30 min. The resulting supernatant was loaded onto a 1 mL HisTrap HP column (GE Healthcare), pre-equilibrated with Buffer A. After washing with 15 mL of Buffer A, protein bound to the column was eluted with linear gradient of imidazole (20–500 mM) generated by mixing Buffer A and Buffer B (20 mM HEPES pH 7.5, 500 mM imidazole, 250 mM NaCl). Sample fractions were analyzed by SDS-polyacrylamide gel electrophoresis (SDS-PAGE), and those containing Cmr-α effector complexes were pooled together, concentrated and further purified by size exclusion chromatography (SEC) in Buffer C (20 mM Tris-HCl pH 8.0, 500 mM NaCl) with a Superdex 200 Hiload column (GE Healthcare, Chicago, IL, USA). All SEC fractions were analyzed by SDS-PAGE, and those containing the complete set of Cmr-α subunits were pooled together, concentrated and stored at −20 °C until use.

### 4.5. Labeling of DNA and RNA Substrates

DNA and RNA substrates used in cleavage assays were 5′ labeled with γ^32^P-ATP using T4 polynucleotide kinase (Thermo Fisher Scientific, Waltham, MA, USA). All substrates were purified by recovering the corresponding bands from a denaturing PAGE gel. DNA and RNA oligonucleotides (Supplementary Table S4) to be used as substrate for cleavage assays were purchased from IDT, USA.

### 4.6. Nucleic Acids Cleavage Assays

RNA/DNA cleavage assays were conducted as previously described [87]. Briefly, reaction mixtures were set up as 10 µL of mixture containing 20 mM MES (pH 6.0), 10 mM MnCl_2_, 5 mM DTT, 50 nM substrate (RNA or DNA) and 50 nM effector complex. Incubation was for the time periods indicated in each assay, and the reaction was then stopped by addition of 2× loading dye (New England Biolabs, Ipswich, MA, USA) and set immediately on ice. Before loading, samples were heated for 5 min at 95 °C, and cleavage products were separated on denaturing polyacrylamide gels (18% polyacrylamide, 40% urea) and visualized by phosphor imaging. 

### 4.7. cOA Synthesis Assay

The synthesis of cOA was conducted as described previously [87]. In short, the reaction mixture (10 µL in total) contained 20 mM MES (pH 6.0), 10 mM MnCl_2_, 5 mM DTT, 100 μM ATP, 200 nM SS1-46 RNA, about 1 nM α^32^P-ATP and 50 nM effector complex. The reaction was performed at 70 °C and stopped at the indicated time point by the addition of 2 × RNA loading dye (New England Biolabs). The cOA products were analyzed on a denaturing 24% (29:1 acrylamide: bis-acrylamide) polyacrylamide gel and visualized by phosphor imaging.

## Data Availability

All data published in this article are accessible upon request.

## Supplementary Materials

The information can be downloaded at: https://www.mdpi.com/article/10.3390/ijms23158515/s1. References [25,58,59,62,66,88,89,90] are cited in the supplementary materials.
