# Supplementary material for: Inactivation of Target RNA Cleavage of a III-B CRISPR-Cas System Induces Robust Autoimmunity in Saccharolobus islandicus
Source: Int J Mol Sci. 2022 Jul 31;23(15):8515. doi: 10.3390/ijms23158515 (PMC9368842; doi:10.3390/ijms23158515)
Supplement: Supplementary file 1 [file ijms-23-08515-s001.zip › ijms-1826332-supplementary.pdf]

Supplementary data

1. Supplementary tables:

Supplementary table S1. ***Sulfolobus*** **strains used in this work**

Strains	Genotype and features	Reference
ΔβE233	Derived from *S. islandicus* Rey15A, carrying deletion of the *pyrEF* genes and the type III-B Cmr-β locus including 7 *cmr-β* genes	(1)
ΔβE233S1	Carrying deletion of the *pyrEF* genes, the *lacS* gene and the type III-B Cmr-β locus including 7 *cmr-β* genes	(2)
ΔαΔβE233S1	Carrying deletion of the *pyrEF* genes, the *lacS* gene, the Cmr-α locus including 6 *cmr-α* genes and the Cmr-β locus including 7 *cmr-β* genes	(2)
ΔarrayΔβE233	Derived from ΔβE233, carrying deletion of two CRISPR loci and the type I-A module	This work
ΔarrayΔβE233S1	Derived from ΔβE233S1, carrying deletion of two CRISPR loci and the type I-A module; equal to MF1	(3)
4α-H16A	Derived from ΔβE233, carrying a single mutation (H16A) of *cmr4α*	This work
4α-D83A	Derived from ΔβE233, carrying a single mutation (D83A) of *cmr4α*	This work
4α-K46/50A	Derived from ΔβE233, carrying a double mutation (K46A, K50A) of *cmr4α*	This work
4α-W197A	Derived from ΔβE233, carrying a single mutation (W197A) of *cmr4α*	This work
4α-E199/Y201A	Derived from ΔβE233, carrying a double mutation (E199A, Y201A) of *cmr4α*	This work
4α-K251A	Derived from ΔβE233, carrying a single mutation (K251A) of *cmr4α*	This work
4α-4G-A	Derived from ΔβE233, carrying a quadruple mutation (G244A, G245A, G250A, G252A) of *cmr4α*	This work
HD^m	Derived from ΔβE233, carrying a double mutation (H14A, D15A) of *cmr2α*	This work
$Palm^m$	Derived from ΔβE233, carrying a double mutation (D667A, D668A) of *cmr2α*	This work
HD^mPalm^m	Derived from ΔβE233, carrying a quadruple mutation (H14A, D15A, D667A, D668A) donor DNA of *cmr2α*	This work
$HD^mPalm^m4\alpha^{D27A}$	Derived from Δβ-HD^mPalm^m, carrying a single mutation (D27A) of *cmr4α*	This work

Supplementary table S2. Plasmids used in this work

Plasmids	Genotype and features	Reference
pSeSD1	A *Sulfolobus-E. coli* shuttle vector with an expression cassette controlled under ParaS-SD promoter	(4)
pSe-Rp	A *Sulfolobus* artificial mini-CRISPR cloning vector	(5)
pAC-SS1	An artifcial mini-CRISPR locus plasmid derived from pSe-Rp, carrying one spacer matching the protospacer 1 (SS1) of the *S. islandicus* lacS gene	(5)
pAC-MS1	Derived from pSeSD1, carrying an artificial CRISPR locus with 10 copies of 43 nt SS1 spacer.	(6)
pAC-cmr6α-10His	Derived from pAC-MS1, both carrying an artificial CRISPR locus with 10 copies of SS1 spacer and expressing His-tagged Cmr6α	(6)
pS10i	An invader plasmid carrying a target sequence of spacer 10 in CRISPR locus 2 in *S. islandicus*	(7)
pSi_1125	A self-targeting plasmid expressing crRNA targeting the *SiRe_1125* (*alba*) gene	This work
pCmr4α	Derived from pSeSD1, expressing His-tagged Cmr4α	This work
pCmr4α-D27A	Derived from pCmr4α with a single mutation(D27A) of the cmr4α gene, expressing His-tagged Cmr4α-D27A	This work
pAC-SS1-Cmr4α	Derived from pAC-SS1, both carrying an artificial CRISPR locus with 1 copy of SS1 spacer and expressing His-tagged Cmr4α	This work
pAC-SS1-Cmr4α-D27A	Derived from pAC-SS1, both carrying an artificial CRISPR locus with 1 copy of SS1 spacer and expressing His-tagged Cmr4α-D27A	This work
pGE-4α-D27A	A genome editing plasmid for single mutation(D27A) of the *cmr4α* gene in *S.islandicus*	This work
pGE-4α-H16A	A genome editing plasmid for single mutation(H16A) of the *cmr4α* gene in *S.islandicus*	This work
pGE-4α-D83A	A genome editing plasmid for single mutation(D83A) of the *cmr4α* gene in *S.islandicus*	This work
pGE-4α-K46/50A	A genome editing plasmid for double mutation (K46A, K50A) of the *cmr4α* gene in *S.islandicus*	This work
pGE-4α-W197A	A genome editing plasmid for single mutation(W197A) of the *cmr4α* gene in *S.islandicus*	This work
pGE-4α-E199/Y201A	A genome editing plasmid for double mutation (E199A, Y201A) of the *cmr4α* gene in *S.islandicus*	This work
pGE-4α-K251A	A genome editing plasmid for single mutation(K251A) of the *cmr4α* gene in *S.islandicus*	This work
pGE-4α-4G-A	A genome editing plasmid for quadruple mutation (G244A, G245A, G250A, G252A) of the *cmr4α* gene in *S.islandicus*	This work
pGE-2α-HD-A	A genome editing plasmid for double mutation (H14A, D15A) of the *cmr2α* gene in *S.islandicus*	This work
pGE-2α-DD-A	A genome editing plasmid for double mutation (D667A, D668A) of the *cmr1α* gene in *S.islandicus*	This work

Supplementary table S3. Oligonucleotides used in this work

Oligonucleotide	Sequence (5'-3')
MRS-up	ATGCCCCGGGATGTTAAACAAGTTAGG
MRS-dw	GGCACTCGAGAAAAAAAAGATTTTGCTTAATGGTG
Si_1125-up	AAAGAATGCAGCTAAGACATAGTTCATTACTGGTTTCTTTCCTA
Si_1125-dw	TAGCTAGGAAAGAAACCAGTAATGAACTATGTCTTAGCTGCATT
Si_1581-up	AAAGATTATATAATTGGATAAGATTTTTCAAAGTATTGTTTTCA
Si_1581-dw	TAGCTGAAAACAATACTTTGAAAAATCTTATCCAATTATATAAT
Cmr4α-fwd	GGGAATTCCATATGACCAAGAGTTATTTAATC
Cmr4α-rev	GTCGTCGACTGAAATCACCTTTATTCT
4α-D27A-SpF	AAAG ACGTGGGAATGGGTAGAGCTCCAGGCGTAGTGGATTTACC
4α-D27A-SpR	TAGC GGTAAATCCACTACGCCTGGAGCTCTACCCATTCCCACGT
4α-D27A-SOEF	GCATGTTGGTATGGGTAGAGCTCCAGGCGTAGTGGCTTTACCGTTC
4α-D27A-SOER	GCCACTACGCCTGGAGCTCTACCCATACCAACATGCACTGGAGTTATTG
4α-D27A-SalIF	ACGCGTCGACTCCAAAGTGTTGAATACC
4α-D27A-NotIR	AAGGAAAAAAGCGGCCGCTTTAATACGTTCCTCGTA
4α-H16A-SpF	AAAG ACGTGGGAATGGGTAGAGCTCCAGGCGTAGTGGATTTACC
4α-H16A-SpR	TAGC GGTAAATCCACTACGCCTGGAGCTCTACCCATTCCCACGT
4α-H16A-SOEF	CAATAACTCCAGTGGCGGTGGGAATGGGTAGAGCT
4α-H16A-SOER	CCCATTCCCACCGCCACTGGAGTTATTGCGTAGGC
4α-H16A-SalIF	ACGC GTCGAC TCCAAAGTGTTGAATACCAT
4α-H16A-NotIR	AAGGAAAAAA GCGGCCGC TTTAATACGTTCCTCGTA
4α-D83A-SpF	AAAG ATCTAGACTGGCAATAGGGTAAAAAACTGGTATGAGATCA
4α-D83A-SpR	TAGC TGATCTCATACCAGTTTTTTACCCTATTGCCAGTCTAGAT
4α-D83A-SOEF	CACTCATACCAGTTTTTTACCCTATTGCCAGCCTTGACGGTTACGTTTATGTTTC
4α-D83A-SOER	ACCGTCAAGGCTGGCAATAGGGTAAAAAACTGGTATGAGTGCAGTAACTATAAAC
4α-D83A-SalIF	ACGC GTCGAC GAAAGTTTTGTCAACTAAGG
4α-D83A-NotIR	AAGGAAAAAA GCGGCCGC AATTCGCTCATGAAATCACC
4α-K46/50A-SpF	AAAG ATGGCTCTAGCTTTAAGGGAGTTTTAAAGTCAACATTAAT
4α-K46/50A-SpR	TAGC ATTAATGTTGACTTTAAAACTCCCTTAAAGCTAGAGCCAT
4α-K46/50A-SOEF	GTATGGCTCTAGCTTTGCAGGAGTTTTAGCATCAACATTAATGAGT
4α-K46/50A-SOER	GATGCTAAAACTCCTGCAAAGCTAGAGCCATACACTATTGGATAA
4α-K46/50A-SalIF	ACGC GTCGAC GATAAGCAGGGAAGAAGGGT
4α-K46/50A-NotIR	AAGGAAAAAA GCGGCCGC GTCCACAAATTTTGAGACGT
4α-W197A-SpF	AAAG AAATTTGTGGACTGAGGAGTATTTACCGCAAGGAACAGTA
4α-W197A-SpR	TAGC TACTGTTCCTTGCGGTAAATACTCCTCAGTCCACAAATTT
4α-W197A-SOEF	AAACGTCACAAAATTTGGCGACTGAGGAGT
4α-W197A-SOER	TCGCCAAATTTTGTGACGTTTTATTTTCAT
4α-W197A-SalIF	ACGC GTCGAC AATAGGTTATCCAATAGTCT
4α-W197A-NotIR	AAGGAAAAAA GCGGCCGC GACCGAAATATTCTTGCCTA
4α-E199/Y201A-SpF	AAAG AAATTTGTGGACTGAGGAGTATTTACCGCAAGGAACAGTA

Oligonucleotide	Sequence (5’-3’)
4α-E199/Y201A-SpR	TAGC TACTGTTCCTTGCGGTAAATACTCCTCAGTCCACAAATTT
4α-E199/Y201A-SOEF	ATAAAAGACAAGGTTTATGTATTTGATAAC
4α-E199/Y201A-SOER	GTTCCTTGCGGTAAAGCCTCTGCAGTCCAC
4α- E199/Y201A-SalIF	ACGC GTCGAC AATAGGTTATCCAATAGTCT
4α- E199/Y201A-NotIR	AAGGAAAAAA GCGGCCGC GACCGAAATATTCTTGCCTA
4α-K251A-SpF	AAAG TAGGAGGTAAGGAAACTATAGGTAAAGGACTTGTAAGAAT
4α-K251A-SpR	TAGC ATTCTTACAAGTCCTTTACCTATAGTTTCCTTACCTCCTA
4α-K251A-SOEF	GTAAGGAAACTATAGGTGCAGGACTTGTAA
4α-K251A-SOER	CCTATAGTTTCCTTACCTCCTAGAAATACA
4α-K251A-SalIF	ACGC GTCGAC GTTTTTTACCCTATTGCCAG
4α-K251A-NotIR	AAGGAAAAAA GCGGCCGC ATTTTCTCACTCATATGGTA
4α-4G-A-SpF	AAAG TAGGAGGTAAGGAAACTATAGGTAAAGGACTTGTAAGAAT
4α-4G-A-SpR	TAGC ATTCTTACAAGTCCTTTACCTATAGTTTCCTTACCTCCTA
4α-4G-A-SOEF	CTAAGGAAACTATAGCTAAAGCACTTGTAA
4α-4G-A-SOER	GCTATAGTTTCCTTAGCTGCTAGAAATACA
4α-4G-A-SalIF	ACGC GTCGAC GTTTTTTACCCTATTGCCAG
4α-4G-A-NotIR	AAGGAAAAAA GCGGCCGC ATTTTCTCACTCATATGGTA
2α-HD-A-SpF	AAAG CGACCCTCCTTGGAAGGCATGGGTAATTACAAGGAATATT
2α-HD-A-SpR	TAGC AATATTCCTTGTAATTACCCATGCCTTCCAAGGAGGGTCG
2α-HD-A-SOEF	GCCTATTTTGCCGCCCCTCCTTGGAAGGC
2α-HD-A-SOER	GGAGGGGCGGCAAAATAGGCTATTATTTTCTTATTTAAGAAC
2α-HD-A-SalIF	ACGC GTCGAC TCTGGCAAGATATTAGAC
2α-HD-A-NotIR	AAGGAAAAAA GCGGCCGC GTGGGGATAAGAAATAAATTAAGTGG
2α-DD-A-SpF	AAAG GTTAAACTTTATAGGCGATAACGCCAATATGTCGTCACCA
2α-DD-A-SpR	TAGC TGGTGACGACATATTGGCGTTATCGCCTATAAAGTTTAAC
2α-DD-A-SOEF	TTGGCGTTATCGCCTATAAAATTCAATGAAAAGTACGTAG
2α-DD-A-SOER	CTTTTCATTGAATTTTATAGGCGATAACGCCAAAATAGCAGCACCACCCAAAT ATATAG
2α-DD-A-SalIF	ACGC GTCGAC TCGGCCTGTAATATCAGTGAC
2α-DD-A-NotIR	AAGGAAAAAA GCGGCCGC GTCATATTCCCCGCTCTT

Supplementary table S4. Nucleic acid substrates used in this work

Name	Sequence (5'-3')	Size (mer)
**RNA**		
SS1-46	UGUUAAGUCUGGUUUCCCUCCAGGGUAUCUAAGCUUUGAAAAAAAA	46
**DNA**		
S10-60	ACTATAGGGAGaATAGAATGCCCCCATTATACAATATCTACGTTTTAGATGAc ccccccc	60

2. Supplementary figures

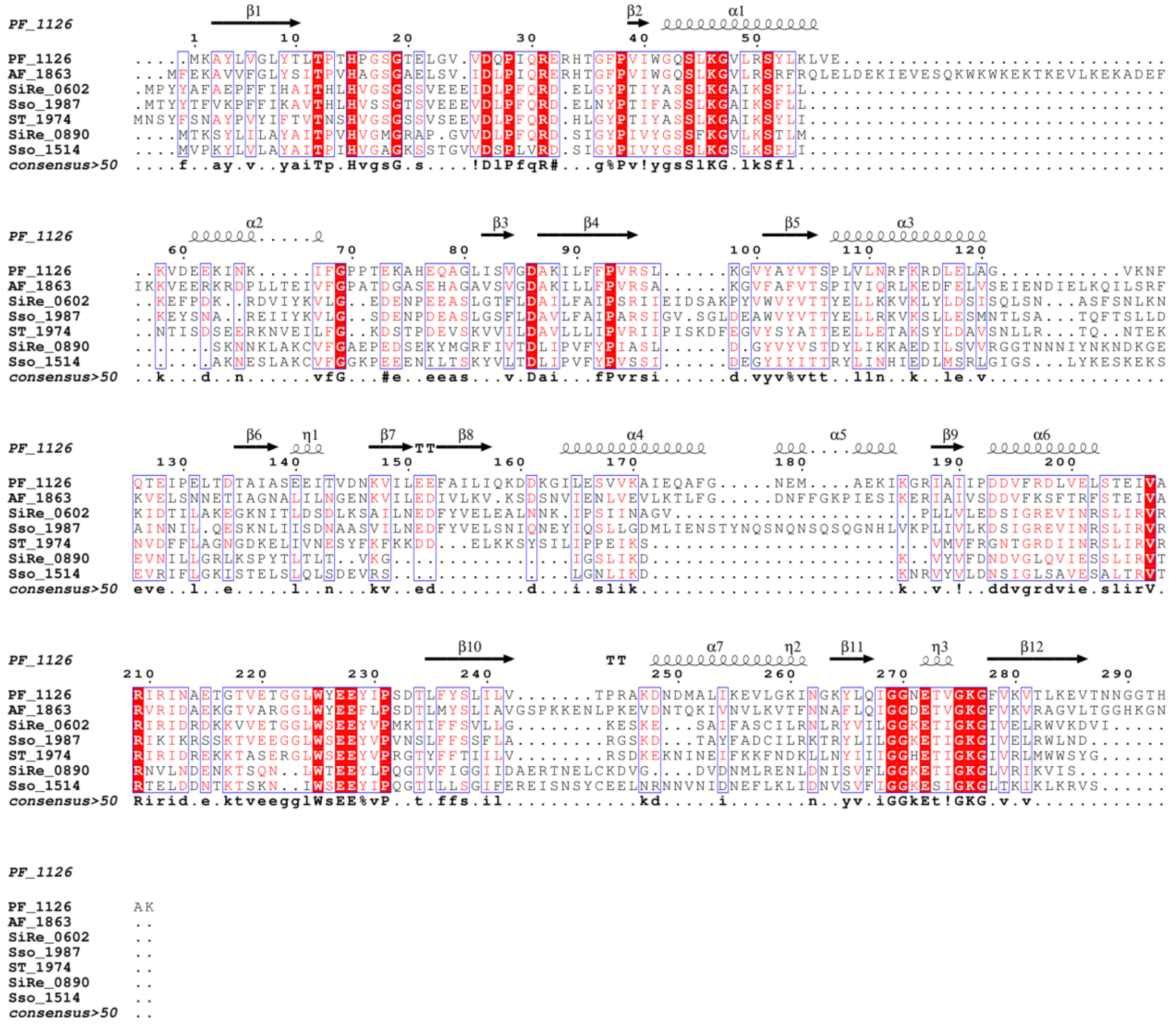

Supplementary figure S1. Conserved residues in *S. islandicus* Cmr4α. Seven Cmr4 homologues (PF_1126, AF_1863, SiRe_0890, SSo1514, SSo_1987, SiRe_0602, ST_1974) were selected and aligned using ESPript 3.x (8). Thirteen highly conserved residues/motifs were chosen to produce eight Cmr4α mutants as indicated below: Cmr4α-H16A, Cmr4α-D27A, Cmr4α-D83A, Cmr4α-K46/50A, Cmr4α-W197A, Cmr4α-E199/Y201A, Cmr4α-K251A and Cmr4α-4G-A.

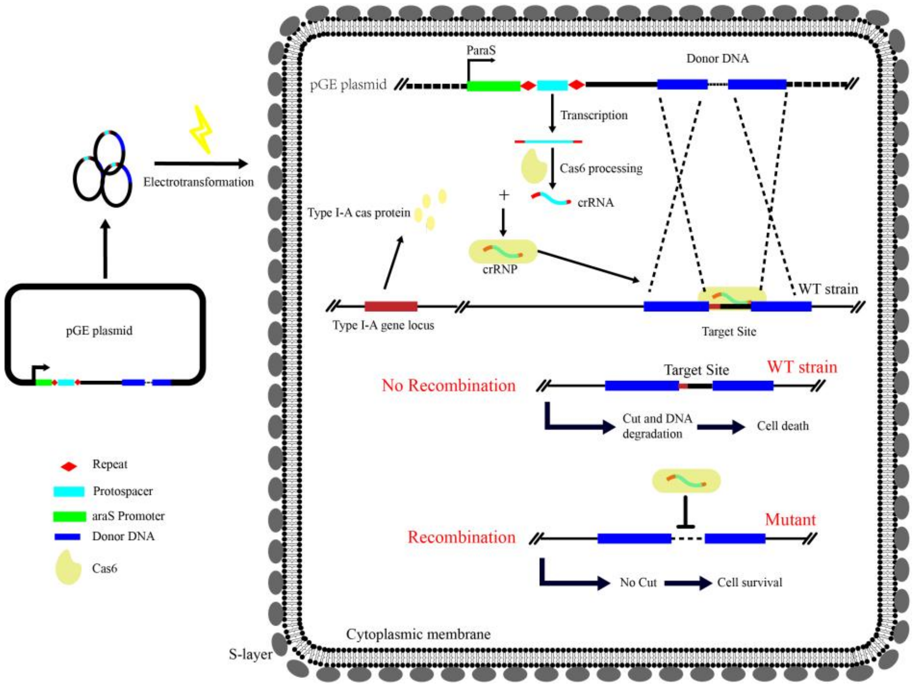

Supplementary figure S2. Mutagenesis was conducted by introduction of pGE plasmids into ΔCmr-β cells by electroporation. The rationale is that crRNAs expressed from the CRISPR array in pGE plasmids guide the endogenous I-A immunity to specifically target individual fragment of the wild-type cmr4α gene for degradation, and in the meantime, the plasmid-borne homologous arms carrying the mutated *cmr4α* gene are allowed to recombine with the wild-type gene, giving the corresponding mutated *cmr4α* gene in the host chromosome.

A

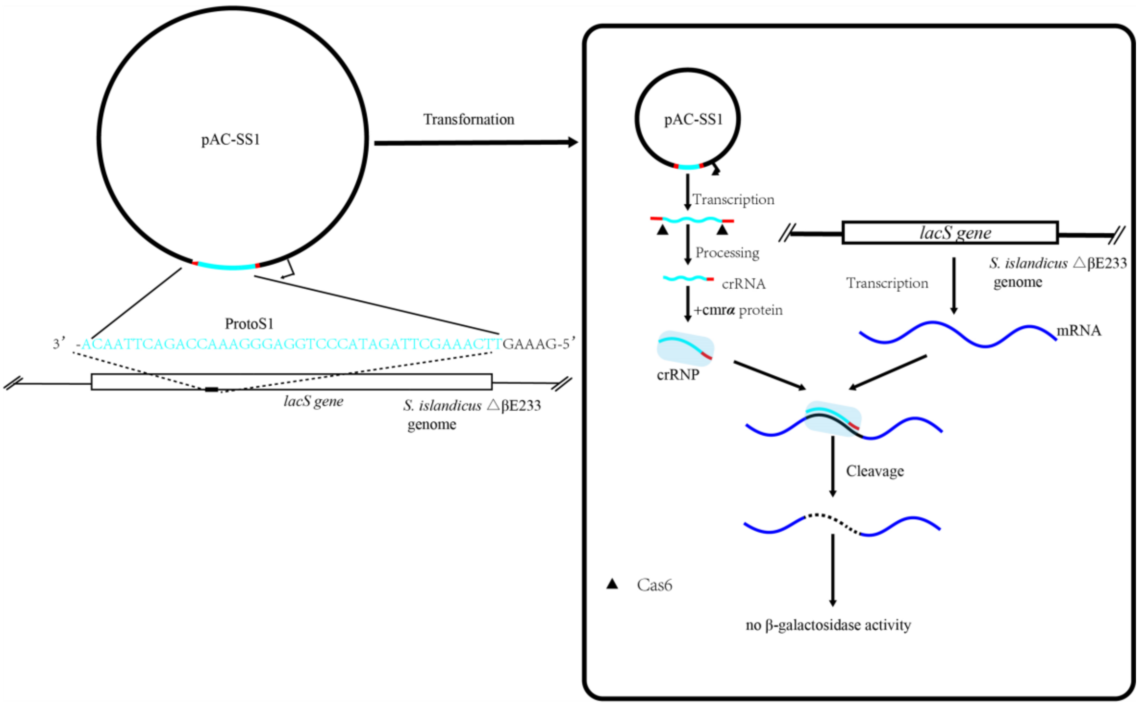

B

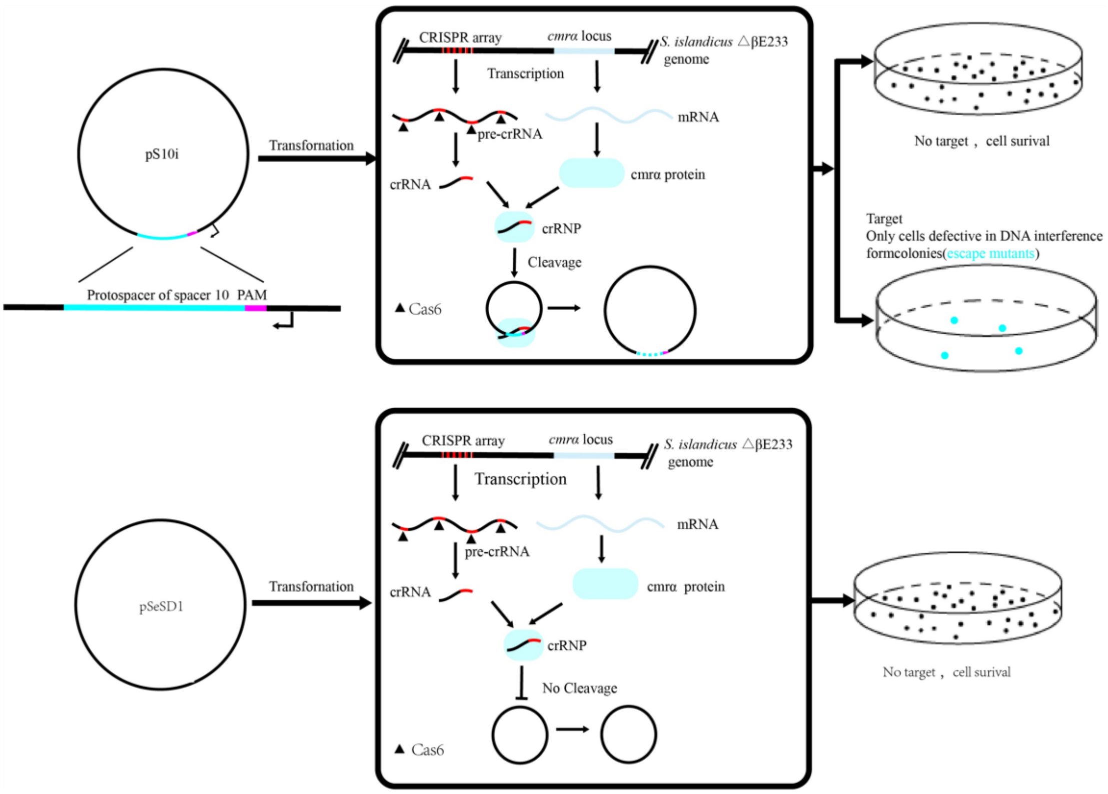

Supplementary figure S3. Two genetic assays developed for *S. islandicus*: the mini-CRISPR and reporter gene-based RNA interference assay (A) and the interference plasmid assay (B)

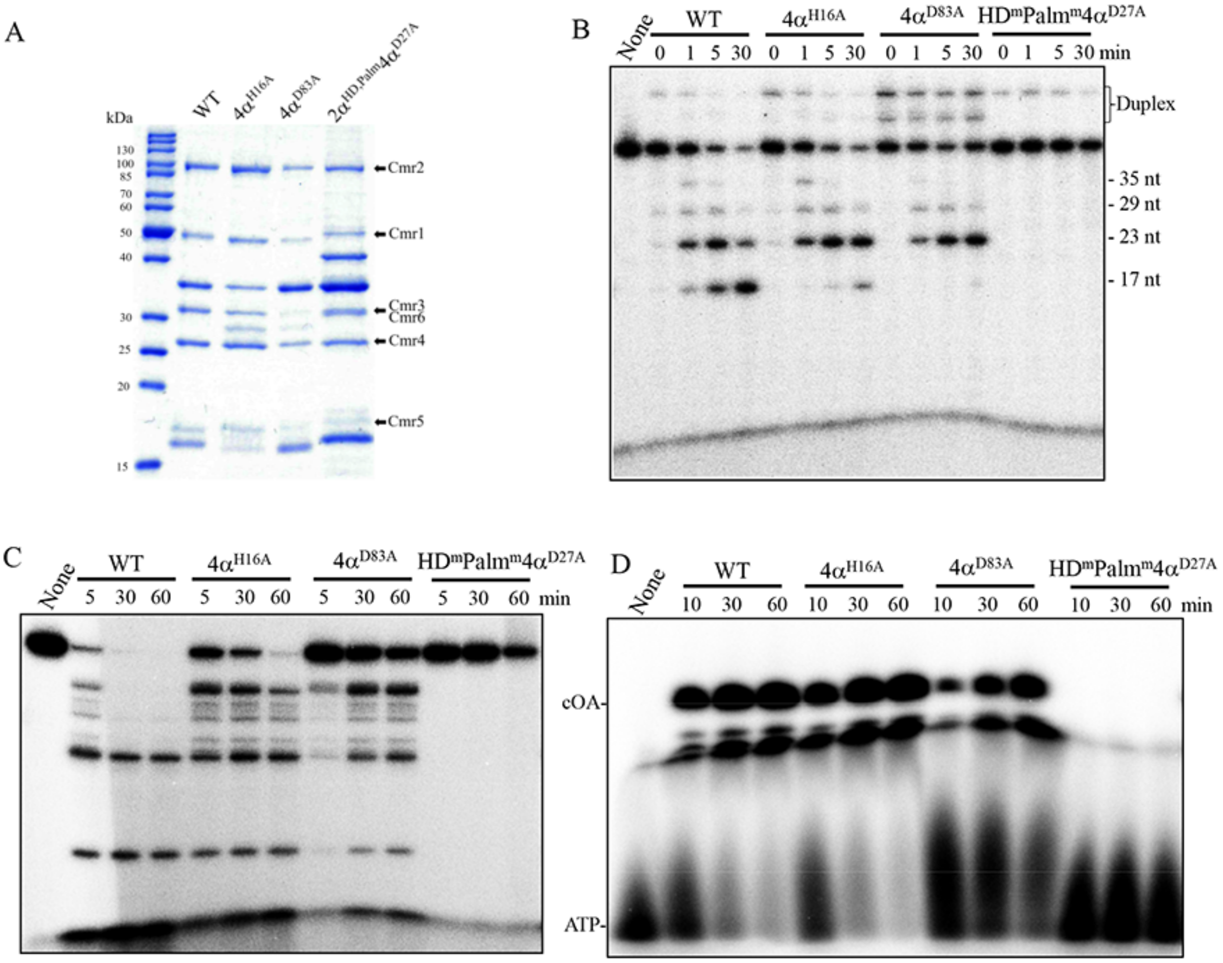

Supplementary figure S4. In vitro activities of wild type (WT) Cmr-α, Cmr-α_$4\alpha^{H16A}$, Cmr-α_$4\alpha^{D83A}$ and Cmr-α_HD^{m},$Palm^{m}4\alpha^{D27A}$. (A) SDS-PAGE analysis, (B) target RNA cleavage, (C) DNA cleavage and (D) cOA synthesis activity of the four complexes. None: Substrate only, no Cmr complex was added. Cleavage assay was conducted for the time periods indicated in the figures; Duplex: duplex of crRNA and substrate; cOA: cyclic tetra-adenylates.

References

1. Li, Y., Pan, S., Zhang, Y., Ren, M., Feng, M., Peng, N., Chen, L., Liang, Y.X. and **She, Q.** (2016) Harnessing Type I and Type III CRISPR-Cas systems for genome editing. *Nucleic acids research*, **44**, e34.
2. Peng, W., Li, H., Hallstrom, S., Peng, N., Liang, Y.X. and She, Q. (2013) Genetic determinants of PAM-dependent DNA targeting and pre-crRNA processing in Sulfolobus islandicus. *RNA biology*, **10**, 738-748.
3. Han, W., Li, Y., Deng, L., Feng, M., Peng, W., Hallstrom, S., Zhang, J., Peng, N., Liang, Y.X., White, M.F. *et al.* (2017) A type III-B CRISPR-Cas effector complex mediating massive target DNA destruction. *Nucleic acids research*, **45**, 1983-1993.
4. Peng, N., Deng, L., Mei, Y., Jiang, D., Hu, Y., Awayez, M., Liang, Y. and **She, Q.** (2012) A synthetic arabinose-inducible promoter confers high levels of recombinant protein expression in

hyperthermophilic archaeon Sulfolobus islandicus. *Applied and environmental microbiology*, **78**, 5630-5637.

5. Peng, W., Feng, M., Feng, X., Liang, Y.X. and **She, Q.** (2015) An archaeal CRISPR type III-B system exhibiting distinctive RNA targeting features and mediating dual RNA and DNA interference. *Nucleic acids research*, **43**, 406-417.
6. Li, Y., Zhang, Y., Lin, J., Pan, S., Han, W., Peng, N., Liang, Y.X. and She, Q. (2017) Cmr1 enables efficient RNA and DNA interference of a III-B CRISPR-Cas system by binding to target RNA and crRNA. *Nucleic Acids Res*, **45**, 11305-11314.
7. Deng, L., Garrett, R.A., Shah, S.A., Peng, X. and She, Q. (2013) A novel interference mechanism by a type IIIB CRISPR-Cmr module in Sulfolobus. *Mol Microbiol*, **87**, 1088-1099.
8. Robert, X. and Gouet, P. (2014) Deciphering key features in protein structures with the new ENDscript server. *Nucleic Acids Res*, **42**, W320-324.
